# PKM2 orchestrates tumor progression via metabolic reprogramming and MDSCs-mediated immune suppression in the tumor microenvironment

**DOI:** 10.3389/fimmu.2025.1588019

**Published:** 2025-08-29

**Authors:** Wenxi Liu, Jiaqi Wu, Xinran Zhang, Yanhua Zhang, Xianqin Zeng, Xiaochun Peng

**Affiliations:** ^1^ Department of Pathophysiology, School of Basic Medicine, Health Science Center, Yangtze University, Jingzhou, China; ^2^ Department of Oncology, First Affiliated Hospital of Yangtze University, Jingzhou, China; ^3^ Department of Gynaecology and Obstetrics, Tongji Medical College, Union Hospital, Huazhong University of Science and Technology, Wuhan, China

**Keywords:** tumor microenvironment, glycolysis, pyruvate kinase M2 type, cysteinecathepsins, cathepsins, myeloid-derived suppressor cells, T cell

## Abstract

The tumor microenvironment (TME) is a complex system, in which the energy metabolism of tumor cells plays a key role in the occurrence, development and metastasis of tumors. In the TME, the energy supply of tumor cells mainly comes from glycolysis. This metabolic reprogramming phenomenon is usually called the Warburg effect. Despite the abundance of oxygen, tumor cells still preferentially utilize the glycolytic pathway to meet their bioenergetic demands. Pyruvate kinase (PK), as a key enzyme in glycolysis, plays an important role in the regulation of energy metabolism in tumor cells. Among them, pyruvate kinase M2 (PKM2) is highly expressed in tumors and promotes the release of cytokines by tumor cells, thereby recruiting myeloid-derived suppressor cells (MDSCs). These cytokines bind to the surface receptors of MDSCs, activate related signaling pathways, and up-regulate the expression of cathepsin cysteine proteases. This process subsequently inhibits the activity of T cells, thereby affecting tumor development.

## 1 Introduction

The occurrence and development of tumors are complex multi-step processes driven by the dysregulation of key molecular mechanisms and signaling pathways. The TME is composed of heterogeneous cellular and non-cellular components, including cancer cells, cancer-associated fibroblasts (CAFs), endothelial cells, various immune cell populations (such as tumor-associated macrophages (TAMs), T cells, and MDSCs, extracellular matrix (ECM), and a range of signaling molecules (cytokines, chemokines, growth factors), often accompanied by abnormal physical conditions (such as hypoxia). This complex environment is not static but is constantly being dynamically remodeled by the interactions between tumor cells and stromal components. These interactions profoundly influence key cancer characteristics, including proliferation, invasion, metastasis, immune evasion, and metabolic adaptation. Emerging evidence highlights the important functional roles of the metabolic enzyme PKM2 and cathepsins in the dynamic TME, particularly in promoting tumor progression. PKM2, as the rate-limiting enzyme in glycolysis, is a central regulator of metabolic reprogramming in tumor cells and can promote the Warburg effect (aerobic glycolysis) even under oxygen-rich conditions (1). This metabolic shift provides the necessary bioenergetic and biosynthetic substrates for the rapid growth and proliferation of tumor cells in the TME (2–4). Cat belong to the lysosomal cysteine protease family and play crucial roles in ECM degradation (facilitating invasion and metastasis), processing of signaling molecules, and, importantly, suppressing the activity of immune cells (particularly T cells). Notably, the influence of PKM2 is not limited to the metabolism of cancer cells. It actively shapes the immunosuppressive characteristics of the TME by promoting the secretion of specific cytokines by tumor cells. These cytokines not only recruit immunosuppressive cell types such as MDSCs but also activate cathepsins within these cells upon binding to their receptors (5–8). Once activated, Cat released by MDSCs (and other TME components) exert powerful immunomodulatory effects, such as suppressing T cells and further promoting immune evasion. This review systematically examines the molecular interactions between PKM2 and cathepsins in MDSCs and particularly emphasizes their synergistic mechanisms in driving tumor progression.

## 2 The structural dynamics of PKM2 regulates the occurrence of tumors

PKM2 maintains dynamic equilibrium between monomeric, dimeric, and tetrameric states. The tetrameric conformation of PKM2 contains a structurally heterogeneous regulatory domain that adopts a seesaw-like allosteric configuration. When some isomeric regulators are inserted into the spatial structure involved in the isomeric regulation of PKM2, the tetrameric PKM2 can change from a compact state (R state) to a loose state (T state), and finally dissociates into a dimer form (9, 10). Dimeric PKM2 exhibits protein kinase activity that orchestrates cellular signaling cascades and epigenetic regulation (11). PKM2 participates in cell signaling and gene regulation due to a nuclear localization signal (NLS) sequence, a 139-amino acid residue segment located in its C domain. This NLS enables PKM2 to translocate to the nucleus under specific conditions, such as in tumor cells. Within the nucleus, PKM2 acts as a transcriptional coactivator and regulates the expression of genes involved in cell growth, proliferation, and differentiation (12, 13). This dual capacity enables PKM2 to simultaneously coordinate metabolic reprogramming and transcriptional activation, thereby supporting rapid cellular proliferation. During embryogenesis, elevated PKM2 activity correlates with enhanced mitotic rates, whereas PKM1 becomes predominant during terminal differentiation (14, 15). Moreover, during tumorigenesis, PKM1 expression is significantly reduced and PKM2 expression is markedly increased, reflecting the high proliferation rate of tumor cells (16, 17). PKM2 overexpression in tumors is associated with poor prognosis in various digestive system malignancies, including gastric cancer, esophageal squamous cell carcinoma, hepatocellular carcinoma, biliary tract cancer, and oral squamous cell carcinoma. High PKM2 expression levels are significantly correlated with reduced overall survival (OS). Furthermore, PKM2 overexpression correlates with adverse clinicopathological features, such as advanced clinical stage, larger tumor size, lymph node metastasis, and poor differentiation. Accumulating evidence establishes PKM2 as a master regulator driving tumor initiation and progression through its pleiotropic functions (18, 19). In addition to the pleiotropic roles of PKM2 in tumor metabolism and transcriptional regulation, Cats also play a significant role in the TME, particularly by modulating MDSCs.

## 3 The structure of cysteine cathepsins and their effects on MDSCs and other immune cells

The structure of Cat usually has a conserved catalytic domain containing cysteine (Cys), histidine (His) and asparagine (Asn) residues, which form the active site of the enzyme. This structure allows them to efficiently catalyze the hydrolysis of peptide bonds, while Cat usually exists in the form of inactive zymogen (20). Following Golgi-mediated post-translational modification, procathepsins are trafficked to lysosomes, where acidic pH induces autocatalytic removal of inhibitory propeptides, yielding mature enzymes with proteolytic competence (21). The proteolytic activity of cysteine cathepsins has been mechanistically linked to tumor progression, particularly through their involvement in myeloid-derived suppressor cell (MDSCs)-mediated immunosuppression (22). In different tumor types, the dependence of MDSCs on cathepsins may vary. For instance, in some solid tumors, MDSCs may rely more on CatS to maintain their immunosuppressive function, while in other tumors, other cathepsins (such as CatL, CatX, etc.) may play a more significant role. Moreover, the expression of Cat in MDSCs is regulated by cytokines, chemokines, and metabolic pathways (23, 24). A total of 11 types of Cat have been found, including cathepsins B, C, F, H, K, L, O, S, V, W and X, and different types of cathepsins have different effects on tumors (25). Some cat, such as cathepsins B, C, F, H, L and O, are ubiquitously expressed in the human body, while others are restricted to specific cells and tissues (26, 27). Cathepsin W (lymphopain) demonstrates cytotoxic cell-specific expression, predominantly localizing to natural killer (NK) cells with limited detection in CD8^+^T lymphocytes (28). Cathepsin K serves as a osteoclast-specific protease critical for bone matrix resorption (29). Cathepsin V, also known as cathepsin L2, is mainly expressed in the cornea, thymus, heart, brain and skin and is involved in the release of antigenic peptides and the maturation of MHC class II molecules (30). Jakoš et al. (31, 32) discovered through experiments that lysosomal cathepsins L/X undergo dramatic upregulation during MDSCs differentiation, concomitant with enhanced proteolytic capacity. In a mouse model of breast cancer metastasis to bone, high levels of Cat were detected in myeloid-derived suppressor cells (MDSCs) of mice with highly metastatic tumors. Among them, cathepsin L and X types increased most significantly. Therefore, in the subsequent part of this article, the effects of cysteine cathepsins on immune cells and tumors are mainly discussed based on these two types. The specific role of Cat in the immune system is shown (**Table 1**).

**Table 1:** Immune effects of cysteine cathepsins.

Cysteine tissue protease name	The immune role played	References
Cathepsin B	It is involved in extracellular matrix protein degradation and stimulates NLRP3 inflamma-some to induce apoptosis and inflammatory response caused by IL-1β production in macro-phages	(33, 34)
Cathepsin C	Activates serine proteinases in cytotoxic T cells, mast cells, and neutrophils, thereby enab-ling these cells to kill pathogens and regulate inflammatory re-sponses	(35, 36)
Cathepsin F	It is mainly related to pro-teasome degradation and auto-phagy. It is also associated with cellular immunity and lipo-protein degradation	(37)
Cathepsin H	It can degrade the extracellular matrix, promote the secretion of pro-inflammatory me-diators such as IL-1β, NO and INF-γ, and also activate the complement system	(38)
Cathepsin K	Mainly expressed in osteo-clasts, it is involved in the degradation of matrix collagen during bone resorption and mediates the inflammatory stress response on bone surface	(39)
Cathepsin L	It can work with MDSCs to inhibit cytotoxic T cell activity and promote tumor invasion and metastasis	(32)
Cathepsin O	It can degrade proteins within cells, participate in the process of protein degradation and recycling, and participate in innate immune responses	(40)
Cathepsin S	Cleaved invariant chain p10, which is capable of assembling the MHC Class II-Ag peptide complex and thus plays an important role in regulating the presentation of MHC Class II surface antigen (Ag) from antigen presenting cells (APC) to T and B cells	(41, 42)
Cathepsin V	Involved in the release of antigenic peptides and matur-ation of MHC Class II molecules, and involved in the turnover of elastin fibril and the cutting of intracellular and extracellular substrates	(43)
Cathepsin W	By interacting with CD25 and cutting CD25, it is involved in processing the IL-2 receptor in the cytoplasm, limiting the activation of STAT5 under IL-2 signaling, thereby inhibiting Foxp3 expression and peri-pheral regulatory T cell function	(44, 45)
Cathepsin X	It plays an important role in intracellular protein de-gradation, T cell migration and adhesion, antigen processing and extracellular matrix re-modeling	(46, 47)

## 4 PKM2 coordinates cytokine and metabolic pathways for MDSCs recruitment and immunosuppression in cancer

**Figure 1:**
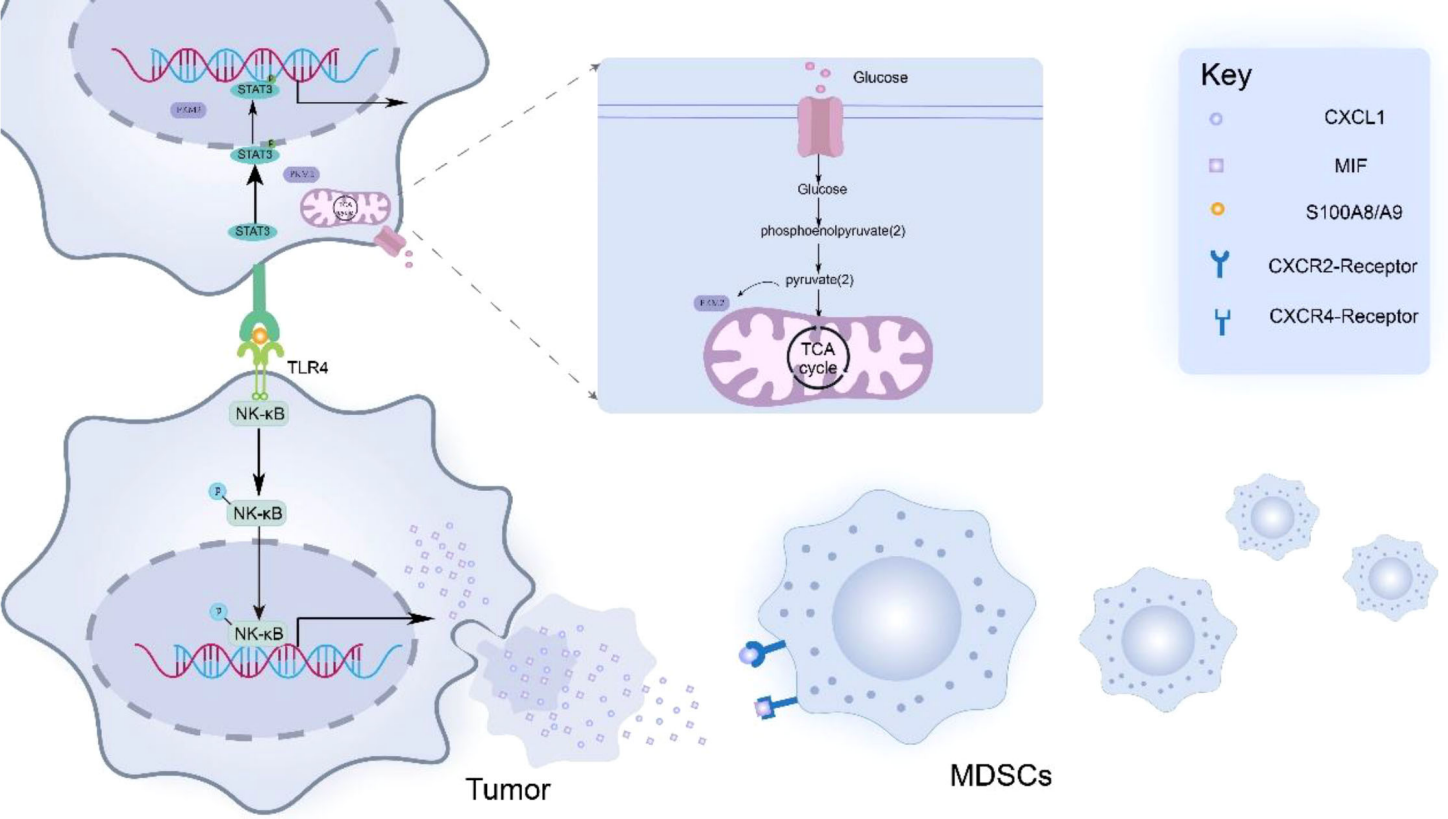
PKM2 promotes tumor cells to secrete cytokines such as MIF and CXCL1, thereby mediating the recruitment and aggregation of MDSCs in the tumor microenvironment. In addition, PKM2 promoted the production of S100 calcium-binding protein A8/A9 (S100A8/A9) in tumor cells. These dimeric proteins then bind to Toll-like receptor 4 (TLR4) expressed on adjacent tumor cells, thereby activating the NF-κB signaling pathway in tumor cells. Activation of the NF-κB signaling pathway leads to the upregulation of MIF and CXCL1 expression, ultimately forming a positive feedback loop that enhances the infiltration of MDSCs into the peritumoral area.

PKM2 promotes cytokine production by tumor cells, which promotes MDSCs infiltration within the TME (48–50). Liu et al. (51) found that by knocking down PKM2, the levels of cytokines (including CCL8, CXCL1, CCL21, CCL2 and MIF) were significantly reduced, thereby confirming the role of PKM2 in cytokine regulation. Specifically, PKM2-induced CXCL1 and MIF produced by tumor cells can bind to CXCR2 and CXCR4 receptors on MDSCs, thereby activating the signaling pathway of MDSCs and triggering cytoskeletal rearrangement, which makes MDSCs chemotactic to the periphery of tumor cells. In addition, the nuclear translocation dimer PKM2 (in a low activity state) acts as a protein kinase and transcriptional coactivator to activate STAT3 signaling pathway in tumor cells. Activation of STAT3 signaling promotes the secretion of S100A8/A9, a heterodimeric calcium-sensing damage-associated molecular pattern (DAMP), which binds to Toll-like receptor 4 (TLR4) on adjacent tumor cells. TLR4 ligation activates NF-κB signaling and upregulates chemokines (e.g., CXCL1, MIF), thereby amplifying MDSC recruitment (52–56) (**Figure 1**).

In addition to coordinating chemokine-driven recruitment, PKM2 further regulates the function and recruitment of myeloid-derived suppressor cells (MDSCs) in the tumor microenvironment (TME) through profound metabolic reprogramming. It is highly expressed in tumor cells, and its dimeric form can act as a coactivator of HIF-1α, promoting the stability of HIF-1α. As a transcription factor, HIF-1α can directly bind to the promoters of various chemokine genes such as CCL2 and CXCL1, thereby upregulating the expression of these chemokines and promoting the recruitment of MDSCs (57). Moreover, the cytokines secreted by recruited MDSCs, such as TGF-β, IL-10, and VEGF, help stabilize HIF-1α, thus forming a positive feedback loop known as the “glycolysis-immunosuppression” circuit (58). After clarifying the mechanisms driving MDSC recruitment and the role of PKM2-induced cytokines, it is necessary to investigate how the binding of these cytokines to their corresponding receptors on MDSCs translates into specific functional consequences. The binding of CXCL1 to CXCR2 and MIF to CXCR4 triggers distinct but crucial intracellular signaling pathways in MDSCs. These pathways converge to induce the expression of Cat (a protease), which is a key mediator of MDSC-mediated immunosuppression.

## 5 Cytokines induce the expression of Cat in MDSCs through associated signaling pathways

In the tumor microenvironment, PKM2 promotes tumor cells to release cytokines and aggregates a large number of MDSCs around tumor cells, PKM2 facilitates the binding of cytokines, such as CXCL1 and MIF, released by tumor cells to the surface receptors CXCR2 or CXCR4 on MDSCs. The interaction between CXCL1 and the receptor CXCR2 activates the STAT3 signaling pathway, phosphorylating STAT3 at tyrosine 705 (Y705) to activate STAT3 signaling (59). After activation of STAT3 signaling pathway, STAT3 can bind to the promoter region of Cat gene as a transcription factor (60–64). Cat gene promoters often contain STAT3 binding sites (65). For example, in some tumor cell studies, STAT3 has been found to bind directly to specific sequence elements in the Cat-B gene promoter and recruit other transcription cofactors, such as histone acetyltransferase (HATs). This complex mediates histone H3K27 and H4K16 acetylation, inducing chromatin relaxation and enhancing transcriptional activation of cathepsin genes (66, 67). Enhanced transcription elevates cathepsin mRNA levels, subsequently boosting protein translation and enzymatic activity.

**Figure 2:**
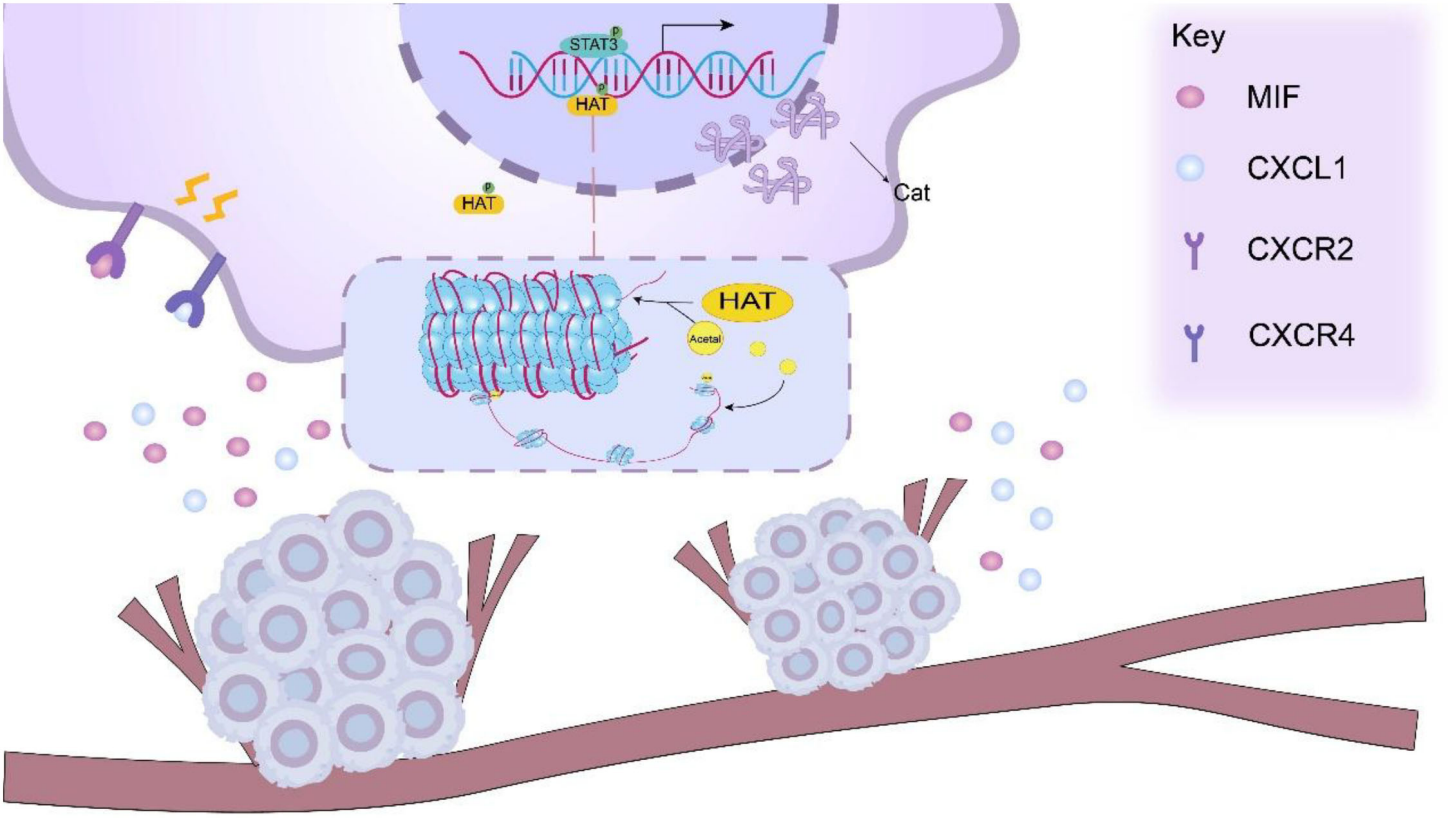
PKM2 facilitates the release of cytokines, such as CXCL1 and MIF, from tumor cells, which can bind to the CXCR2/CXCR4 receptors on the surface of MDSCs. This interaction activates relevant signaling pathways within MDSCs, including the STAT3 signaling pathway. Upon activation of the STAT3 signaling pathway. Activated STAT3 functions as a transcription factor that binds to cathepsin (Cat) gene promoters and recruits transcriptional coactivators including histone acetyltransferases (HATs). HAT-mediated histone acetylation induces chromatin relaxation, thereby potentiating cathepsin gene transcription.

The MIF factor released by tumor cells due to PKM2 can bind to the CXCR4 receptor on the surface of MDSCs and activate the MAPK pathway, thereby initiating the MEK-ERK signaling cascade (68). Activated MAPK signaling facilitates nuclear translocation of ERK, which phosphorylates transcription factors including AP-1 complexes (c-Fos/c-Jun) to enhance their DNA-binding capacity. These transcription factors can bind to the promoter region of cysteine cathepsin related genes to promote gene transcription, thereby increasing the expression of cysteine cathepsin (69–72). Jakošt et al. (32) found that Cat was mainly L and X types in MDSCs, and inhibition of CAT-L was found to restore T cell activity, reflecting that CAT-L could inhibit T cell activity. Janko Kos et al. (73) found that Cat-X affected T cell immune functions, such as migration and adhesion **Figure 2**.

## 6 Cathepsins (L & X) impair T cell function

### 6.1 Effect of Cat-L on T cells

**Figure 3:**
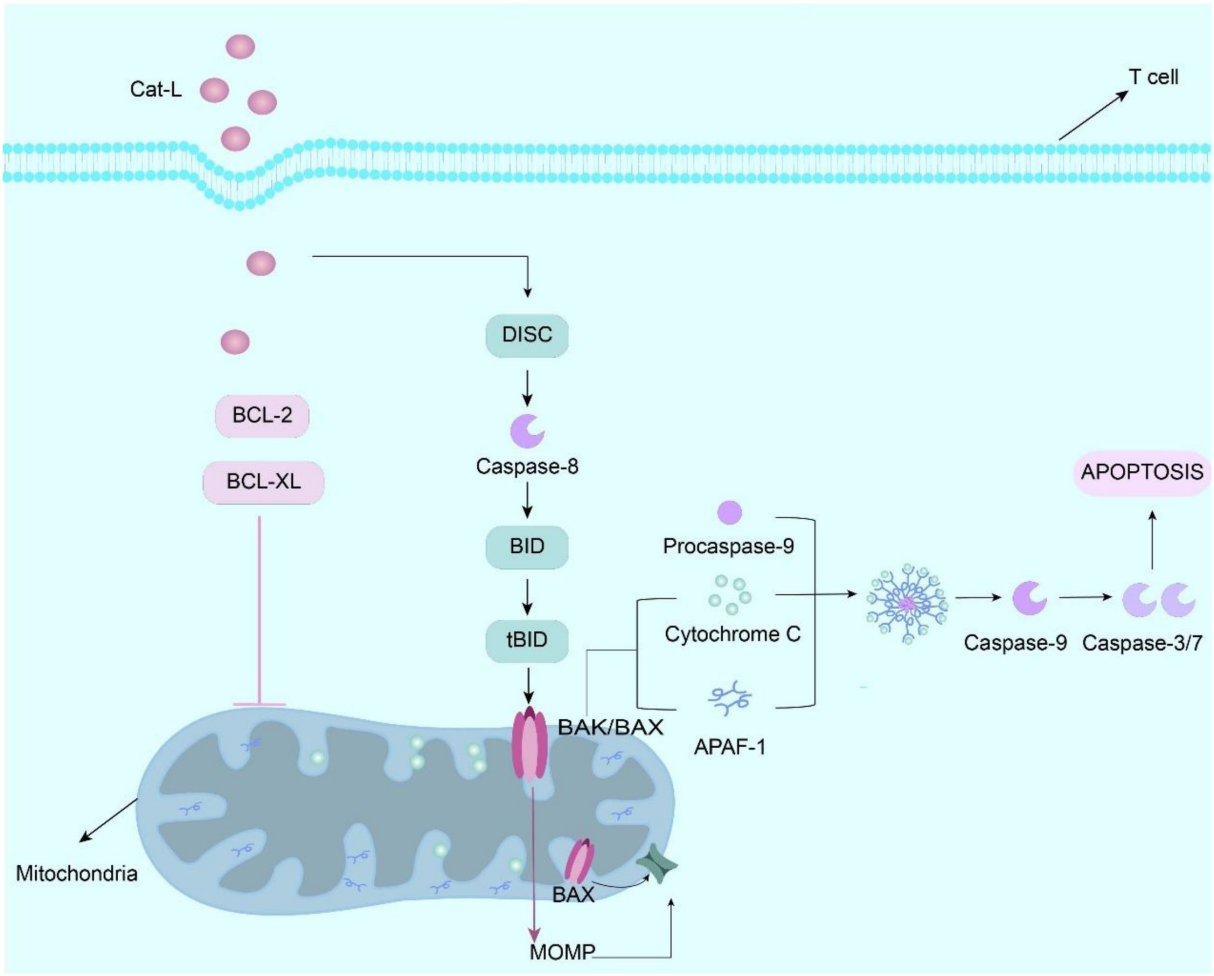
Cathepsin L (Cat-L) is internalized by T cells via receptor-mediated endocytosis, whereupon it proteolytically cleaves Bcl-2 and Bcl-XL, thereby relieving the inhibitory constraint on Bak/Bax dimerization. This triggers mitochondrial outer membrane permeabilization (MOMP), resulting in cytochrome c efflux that complexes with apoptotic protease-activating factor 1 (Apaf-1) to form the apoptosome. The apoptosome recruit procaspase-9 through CARD-CARD interactions, inducing its proteolytic autoactivation into mature caspase-9. Activated caspase-9 proteolytically processes executioner caspases (e.g., caspase-3/7) that systematically dismantle cellular components via cleavage of structural proteins and nucleases, ultimately executing T cell apoptosis. Effect and mechanism of PKM2 on MDSCs.

Emerging evidence indicates that cathepsin L (Cat-L) impairs CD8^+^T cell effector functions, significantly attenuating their tumoricidal activity within the TME (74). As the pore-forming effector protein of cytotoxic lymphocytes (CTLs/NK cells), perforin mediates target cell lysis by facilitating granzyme delivery. Following membrane pore formation, perforin enables granzyme translocation into target cells, initiating caspase-dependent apoptotic cascades. Proteolytic maturation of perforin precursor is essential for acquiring lytic competence, a process tightly regulated in cytotoxic granule (67, 75). Cat-L orchestrates multifaceted immunosuppressive effects via distinct molecular pathways. Mechanistically, Cat-L induces T cell apoptosis through mitochondrial pathway activation. Under normal conditions, anti-apoptotic proteins such as Bcl-2 prevent the increase in mitochondrial membrane permeability and thus inhibit the release of apoptotic factors such as cytochrome C, but Cat-L is able to degrade anti-apoptotic proteins in the Bcl-2 family, such as Bcl-2 and Bcl-XL, Thus, the activation of pro-apoptotic proteins (such as Bad, Bim, Bax, etc.) causes the mitochondria to release cytochrome C and activate the caspase cascade, thereby triggering T cell apoptosis (**Figure 3**) (67, 75). In addition, in the study of the interaction between MDSCs and tumor cells, inhibition of Cat-L activity enhanced the cytotoxicity of CD8^+^ T cells, indicating that Cat-L may have an inhibitory effect on T cell activity under normal conditions, and this inhibitory effect is closely related to the cell-to-cell interaction and microenvironment. Thus, the anti-tumor immune response of the body is affected (31).

### 6.2 Effect of Cat-X on T cells

Concomitant Cat-X upregulation is observed in MDSCs, suggesting coordinated protease-mediated immunosuppression. Cat-X impairs T cell homing via proteolytic cleavage of β2 integrin’s extracellular domain, compromising its lymphocyte adhesion functions essential for immune surveillance. β2 integrin processing by Cat-X initiates signaling cascades that dysregulate talin binding and LFA-1 activation. For example, it will regulate the binding ability of Cat-X to talin, and then affect the affinity of lymphocyte function-associated antigen-1 (LFA-1). This regulatory effect may affect the adhesion ability of T cells, thereby affecting the immune response of T cells (76–79). Cat-X degrades T cell chemo attractants including CXCL12, thereby disrupting CXCR4-mediated chemotactic migration toward inflammatory loci. If CCXL-12 is excessively depleted, it will affect the ability of T cells to migrate (80). Cat-X, as a cysteine protease with exopeptidase activity, is capable of degrading components of the extracellular matrix (ECM) This degradation can reshape the migration pathway of T cells, which in turn affects their migration ability. In the tumor microenvironment, when Cat-X activity is too high, some proteins involved in actin fiber assembly may be over-activated, resulting in excessive polymerization of actin fibers, which makes T cell pseudopods rigid and unable to extend and contract flexibly, and seriously hinders the migration of T cells. The impairment of this migration ability will directly affect the efficacy of T cells in the immune response, making it difficult for T cells to quickly reach the site of infection or tumor tissue, thereby weakening the immune defense ability of the body (81, 82). Cat-X disrupts immunological synapse formation between T cells and antigen-presenting cells (APCs) by modulating surface receptor dynamics. Normally, T cells activate immune responses by recognizing antigen peptide-MHC complexes presented by APCs. Cat-X may alter the expression or function of T cell surface receptors and affect their effective binding and signaling with APC, thereby interfering with T cell recognition of antigens and the initiation of immune responses, thereby promoting tumor progression (83, 84).

## 7 Effects of PKM2 and Cat-L/X on tumors

### 7.1 Effect of PKM2 on tumors

#### 7.1.1 The metabolic regulatory role of PKM2 in tumors

To meet bioenergetic demands, tumor cells preferentially engage in aerobic glycolysis (Warburg effect) despite sufficient oxygen availability. Due to their accelerated proliferation, tumor cells exhibit heightened anabolic requirements: lipids for membrane biogenesis; glucose-derived carbons for protein glycosylation; nucleotide precursors for genome duplication; ribosomal RNA for proteome expansion (85). Within the TME, PKM2 serves as a metabolic orchestrator that channels glycolytic intermediates into proliferative biosynthetic pathways.

As the terminal glycolytic enzyme, PKM2 catalyzes the rate-limiting conversion, generating ATP while maintaining carbon flux essential for tumor biomass accumulation (86). As an important regulator of aerobic glycolysis pathway, PKM2 can provide intermediate metabolites to support the biosynthesis of rapidly dividing cells and avoid oxidative stress damage in tumor cells. Notably, nuclear-translocated PKM2 functions as both a transcriptional coactivator and a protein kinase, directly modulating oncogene expression programs (87). The dimeric PKM2 isoform redirects glucose carbons through branching pathways: (i) lactate/pyruvate generation via glycolysis; (ii) nucleotide/phospholipid synthesis via the pentose phosphate pathway (PPP). Phospholipid biosynthesis, in particular, sustains membrane expansion during neoplastic proliferation and confers structural/functional plasticity to malignant cells (88, 89).

#### 7.1.2 PKM2 drives tumor proliferation

Although PKM2 is much less active than PKM1, PKM2 is able to rapidly perform glycolysis to supply energy to tumor cells (90). Reversible dimer-tetramer transitions enable PKM2 to dynamically regulate glycolytic flux in response to tumor microenvironmental cues. When PKM2 is in the dimeric state, it is unable to efficiently convert phosphoenolpyruvate (PEP) to pyruvate, which leads to the accumulation of glycolytic intermediates. These intermediates can enter the anabolic pathway and be used for the synthesis of biological macromolecules such as nucleic acids and amino acids, which provide the necessary material basis for the rapid growth of tumor cells (91, 92). Wang et al. (92) demonstrated that PKM2 knockdown markedly suppresses tumor growth *in vivo*, validating its non-redundant role in tumorigenesis. Collectively, PKM2 emerges as a pleiotropic oncoprotein that coordinates pro-tumorigenic processes ranging from metabolic adaptation to metastatic dissemination.

### 7.2 Effect of Cat-L/X on tumors

#### 7.2.1 Effect of Cat-L on tumors

Cat-L is highly upregulated in many highly aggressive cancer cell lines and malignancies and is secreted into the extracellular matrix as a precursor enzyme (22). The remodeling of the extracellular matrix has been proven to be crucial for the invasion and migration of tumor cells. For example, in ovarian cancer, when Cat-L is activated, it will degrade various components of the extracellular matrix (such as collagen) to create a pathway for the metastasis of ovarian cancer cells (93). Additionally, Cat-L can also cleave specific proteolytic substrates. For instance, Cat-L can cleave the CDP/Cux protein in the TME, thereby enhancing its ability to activate the VEGF-D gene for transcriptional activation. When VEGF-D expression increases, it promotes the formation of blood vessels and lymphatic vessels around ovarian cancer. This process provides more nutritional supply and migration pathways for ovarian cancer cells. These mechanisms also jointly promote tumor growth, invasion, and metastasis (19, 94). Angiogenesis not only provides nutrients and oxygen but also establishes channels for the spread of tumor cells. Cat-L may also enhance the anti-tumor effect of chemotherapy, radiotherapy, and other treatment methods by weakening the responsiveness to treatment (95). This effect may be attributed to the role of Cat-L in key tumor cell survival mechanisms, including activating anti-apoptotic pathways, thereby promoting immune escape (96). Therefore, Cat-L exerts multifaceted carcinogenic effects through different molecular pathways.

#### 7.2.2 Effect of Cat-X on tumors

As a cysteine protease with terminal protease activity, Cat-X promotes tumor development by degrading extracellular matrix proteins, thereby helping tumor cells break through the basement membrane and enter surrounding tissues and blood vessels, thereby facilitating tumor metastasis. Secondly, in glioblastoma, Cat-X can also regulate intercellular signal transduction in the tumor microenvironment by processing cytokines, chemokines, and cell adhesion molecules. Specifically, it may activate the TGF-β signaling pathway to promote epithelial-mesenchymal transition (EMT), which is a key process for tumor cells to acquire invasive ability (97–99). In addition to promoting tumor cell invasion and metastasis, Cat-X may also help tumor cells evade immune surveillance by regulating immune cell functions in the tumor microenvironment. For example, by degrading the extracellular matrix and modulating the cytokine network, Cath-X may inhibit T cell infiltration and function, thereby promoting tumor immune escape (100). When Cat-B activity is inhibited, Cat-X activity and protein levels are significantly increased to compensate for the loss of Cat-B function. This compensatory mechanism may help to maintain the proteolytic demand of tumor cells and promote tumor growth and invasion (101). Mitrović et al. (37) discovered in *in vitro* cell experiments and *in vivo* tumor mouse models that simultaneously inhibiting cathepsin B and X might significantly affect the progression of tumors.

## 8 Summary and prospect

In tumor biology, PKM2 is overexpressed in tumors and serves as a central metabolic regulator through its dual roles in metabolic reprogramming and gene regulation. PKM2 exhibits diverse functional roles in tumor biology. For example, PKM2 can participate in the process of glucose metabolism as a pyruvate kinase. By regulating glycolytic flux, PKM2 coordinates cancer cell energy metabolism to meet biosynthetic demands during rapid proliferation. PKM2 provides essential material and energy resources to support cancer cell proliferation. PKM2 driven MDSCs recruitment remodels the tumor immune microenvironment, suppressing anti-tumor immunity and facilitating immune escape. Secondly, the cytokines released by tumor cells due to PKM2 can also activate the expression of Cat in MDSCs. Activated Cat can inhibit the activity of CD8^+^T cells, which are the key effector cells of anti-tumor immunity. After its activity is inhibited, the immune surveillance and killing ability of tumor cells is decreased. In addition to inhibiting the activity of T cells, Cat can also affect the migration and adhesion ability of T cells, thereby indirectly promoting the occurrence, development and metastasis of tumors.

To sum up, given the central role of the PKM2-Cat axis in tumor-related processes, they are regarded as highly potential therapeutic targets for a variety of cancers and other metabolic diseases. By inhibiting the activities of PKM2 and Cat, it is expected to break the abnormal metabolic patterns and immune escape mechanisms of tumor cells, opening up new avenues for the treatment of cancer and related metabolic diseases, the current clinical studies related to PKM2 and Cat are shown in (**Table 2**) below. However, due to the complexity and intercorrelation of cellular functions in the body, whether the inhibition of PKM2 and Cat will have a negative impact on the physiological functions of other normal cells in the body, such as interfering with the metabolic balance of normal cells and affecting the normal defense function of the immune system, remains to be further studied in depth.

**Table 2:** The clinical application of PKM2/Cat treatment.

Intervention treatment	Study type	Study overview	Stage	State	Numbering
Fluorine F 18 DASA-23 Pyruvate	Interventional	[18F] DASA-23, is injected into a vein, and a scanner is used to make detailed, computerized pictures of areas inside the body where the sub- stance is used	Phase1	Terminated	NCT03539731
Pleural biopsy	Observational	IP3R Modulation by Cancer Genes Bcl-2 & PKM 2 in Mesothelioma	Unknown status	Unknown status	NCT03558932
Pyruvate kinase isoform M2	Observational	Determine if pyruvate kinase M2 (PKM2) can be used as a biomarker in cancer	Completed	Completed	NCT01130584
ProAgio	Interventional	ProAgio in participants with advanced solid tumor malignancies including pancreatic cancer	Phase1	Active, not recruiting	NCT05085548
ProAgio, Gemcitabine, nab paclitaxel	Interventional	ProAgio combined with gemcitabine and nab paclitaxel (G-nP) in Previously un treated subjects with metastatic pancreatic ductal adeno- carcinoma (PDAC)	Phase1	Recruiting	NCT06182072
Pyruvate kinase isoform M2	Observational	PKM2 has been reported to be associated with tumor progression and some specific tissues and promotes the Warburg effect in cancer cells	Unknown status	Unknown status	NCT01968928
Clarithromycin, Metronidazole, proton pump inhibit	Observational	Investigate the possibility whether strainedpendent differences in Helicobacter pylori lipopolysaccharide (LPS) influence the CTSX expression and cytokine secretion	Unknown status	Unknown status	NCT01137942

## Glossary

APAF-1: Apoptotic protease activator 1
APCs: Antigen-presenting cells
BAX: Bcl-2-associated X protein
Bcl: B-cell lymphoma 2 protein
Bcl-X: B-cell lymphoma X protein
BID: BH3 Interacting Domain Death Agonist
Cat: Cathepsins
CXCL12: C-X-C motif ligand 12
CCL8: C-C motif ligand 8
CCL21: C-C motif ligand 21
CCL2: C-C motif ligand 2
CXCL1: C-X-C motif ligand 1
CXCR2: C-X-C motif receptor 2
CXCR4: C-X-C motif receptor 2
CD8^+^T: Cytotoxic T Lymphocytes
DISC: Death-inducing signaling complex
ERK: Extracellular Signal-Regulated Kinase
ECM: Extracellular matrix
GLUT1: Glucose Transporter 1
H3K2: Histone H3 lysine 27
H4K16: Histone H4 lysine 16
HIF: Hypoxia-inducible factor
HAT: Histone acetyltransferase
IGF1: Insulin-like Growth Factor 1
LDHA: Lactate Dehydrogenase A
MAPK: Mitogen-Activated Protein Kinase
MEK: Mitogen-Activated Protein Kinase Kinase
MOMP: Mitochondrial outer membrane permeability
MIF: Macrophage migration inhibitory factor
MDSCs: Myeloid-derived suppressor cells
NF-κB: Nuclear Factor kappa-light-chain-enhancer of activated B cells
PKM: Pyruvate kinase
PDK: Pyruvate dehydrogenase kinase
STAT3: Signal Transducer and Activator of Transcription 3
S100A8/A9: S100 calcium-binding protein
TIM: Tumor microenvironment
TCA: Cycle Tricarboxylic acid cycle
TLR4: Toll-like receptor4

## References

[1] Wang Y Shu H Qu Y Jin X Liu J Peng W. PKM2 functions as a histidine kinase to phosphorylate PGAM1 and increase glycolysis shunts in cancer. EMBO J. (2024) 43:2368–96. doi: 10.1038/s44318-024-00110-8, PMID: 38750259 PMC11183095

[2] Tamada M Suematsu M Saya H. Pyruvate kinase M2: multiple faces for conferring benefits on cancer cells. Clin Cancer Res. (2012) 18:5554–61. doi: 10.1158/1078-0432.CCR-12-0859, PMID: 23071357

[3] Warburg O. On the origin of cancer cells. Science. (1956) 123:309–14. doi: 10.1126/science.123.3191.309, PMID: 13298683

[4] Zhou H Wang X Mo L Liu Y He F Zhang F. Role of isoenzyme M2 of pyruvate kinase in urothelial tumorigenesis. Oncotarget. (2016) 7:23947–60. doi: 10.18632/oncotarget.8114, PMID: 26992222 PMC5029676

[5] Lv T Fan R Wu J Gong H Gao X Liu X. Tumor-associated macrophages: key players in the non-small cell lung cancer tumor microenvironment. Cancer Med. (2025) 14:e70670. doi: 10.1002/cam4.70670, PMID: 39927632 PMC11808749

[6] Wang P Sun C Zhu T Xu Y. Structural insight into mechanisms for dynamic regulation of PKM2. Protein Cell. (2015) 6:275–87. doi: 10.1007/s13238-015-0132-x, PMID: 25645022 PMC4383751

[7] Wang Y Ding Y Guo N Wang S. MDSCs: key criminals of tumor pre-metastatic niche formation. Front Immunol. (2019) 10:172. doi: 10.3389/fimmu.2019.00172, PMID: 30792719 PMC6374299

[8] Yang L DeBusk LM Fukuda K Fingleton B Green-Jarvis B Shyr Y. Expansion of myeloid immune suppressor Gr+CD11b+ cells in tumor-bearing host directly promotes tumor angiogenesis. Cancer Cell. (2004) 6:409–21. doi: 10.1016/j.ccr.2004.08.031, PMID: 15488763

[9] Morgan HP O’Reilly FJ Wear MA O’Neill JR Fothergill-Gilmore LA Hupp T. M2 pyruvate kinase provides a mechanism for nutrient sensing and regulation of cell proliferation. Proc Natl Acad Sci U.S.A. (2013) 110:5881–6. doi: 10.1073/pnas.1217157110, PMID: 23530218 PMC3625322

[10] Wu B Liang Z Lan H Teng X Wang C. The role of PKM2 in cancer progression and its structural and biological basis. J Physiol Biochem. (2024) 80:261–75. doi: 10.1007/s13105-024-01007-0, PMID: 38329688

[11] Hoshino A Hirst JA Fujii H. Regulation of cell proliferation by interleukin-3-induced nuclear translocation of pyruvate kinase. J Biol Chem. (2007) 282:17706–11. doi: 10.1074/jbc.M700094200, PMID: 17446165

[12] Anastasakis DG Apostolidi M Garman KA Polash AH Umar MI Meng Q. Nuclear PKM2 binds pre-mRNA at folded G-quadruplexes and reveals their gene regulatory role. Mol Cell. (2024) 84:3775–3789.e6. doi: 10.1016/j.molcel.2024.07.025, PMID: 39153475 PMC11455610

[13] Gu J Li X Zhao L Yang Y Xue C Gao Y. The role of PKM2 nuclear translocation in the constant activation of the NF-κB signaling pathway in cancer-associated fibroblasts. Cell Death Dis. (2021) 12:291. doi: 10.1038/s41419-021-03579-x, PMID: 33731686 PMC7969736

[14] Wu M Jia G Liu Y Lou Y Li Y Xia M. PKM2 controls cochlear development through lactate-dependent transcriptional regulation. Proc Natl Acad Sci U.S.A. (2025) 122:e2410829122. doi: 10.1073/pnas.2410829122, PMID: 39773029 PMC11745320

[15] Yang J Wu G Chen H Cai F He S. Effects of M2-type pyruvate kinase on glucose metabolism and growth of bladder cancer cells. J Clin Military Med. (2024) 52:1160–7. doi: 10.16680/j.1671-3826.2024.11.13

[16] Li Y Sun G Li J. The role of PKM2 in tumor metabolism and progression. J Anhui Med Univ. (2018) 53:818–21. doi: 10.19405/j.cnki.issn1000-1492.2018.05.035

[17] Zhu Z Chen M Zhang X Yang Y Zou X. The role of M2-type pyruvate kinase in tumor metabolism and development. Basic Med Clin Med. (2014) 34:1706–9. doi: 10.16352/j.issn.1001-6325.2014.12.033

[18] Jiang Y Wang T Sheng D Han C Xu T Zhang P. Correction: Aurora A-mediated pyruvate kinase M2 phosphorylation promotes biosynthesis with glycolytic metabolites and tumor cell cycle progression. J Biol Chem. (2022) 298:102693. doi: 10.1016/j.jbc.2022.102693, PMID: 36417819 PMC9679659

[19] Turk V Stoka V Vasiljeva O Renko M Sun T Turk B. Cysteine cathepsins: From structure, function and regulation to new frontiers. Biochim Biophys Acta Proteins Proteom. (2012) 1824:68–88. doi: 10.1016/j.bbapap.2011.10.002, PMID: 22024571 PMC7105208

[20] Chitsamankhun C Siritongtaworn N Fournier BPJ Sriwattanapong K Theera- panon T Samaranayake L. Cathepsin C in health and disease: from structural insights to therapeutic prospects. J Transl Med. (2024) 22:777. doi: 10.1186/s12967-024-05589-7, PMID: 39164687 PMC11337848

[21] Pogorzelska A Żołnowska B Bartoszewski R. Cysteine cathepsins as a prospective target for anticancer therapies-current progress and prospects. Biochimie. (2018) 151:85–106. doi: 10.1016/j.biochi.2018.05.023, PMID: 29870804

[22] Vidak E Javoršek U Vizovišek M Turk B. Cysteine cathepsins and their extracellular roles: shaping the microenvironment. Cells. (2019) 8:264. doi: 10.3390/cells8030264, PMID: 30897858 PMC6468544

[23] Fuchs N Meta M Schuppan D Nuhn L Schirmeister T. Novel opportunities for cathepsin S inhibitors in cancer immunotherapy by nanocarrier-mediated delivery. Cells. (2020) 9:2021. doi: 10.3390/cells9092021, PMID: 32887380 PMC7565055

[24] David T du Roure PD Mallavialle A Laurent-Matha V Roger P Guiu S. Cathepsins: Novel opportunities for antibody therapeutics in cancer. Br J Pharmacol. (2025) 182:1671–82. doi: 10.1111/bph.17437, PMID: 39834229

[25] Akiyama M. Cathepsin and cutaneous disorders of cornification and inflammation: their close links. Br J Dermatol. (2023) 189:256–7. doi: 10.1093/bjd/ljad190, PMID: 37287341

[26] Jiang H Dong Z Xia X Li X. Cathepsins in oral diseases: mechanisms and therapeutic implications. Front Immunol. (2023) 14:1203071. doi: 10.3389/fimmu.2023.1203071, PMID: 37334378 PMC10272612

[27] Wex T Bühling F Wex H Günther D Malfertheiner P Weber E. Human cathepsin W, a cysteine protease predominantly expressed in NK cells, is mainly localized in the endoplasmic reticulum. J Immunol. (2001) 167:2172–8. doi: 10.4049/jimmunol.167.4.2172, PMID: 11490002

[28] Brömme D Lecaille F. Cathepsin K inhibitors for osteoporosis and potential off-target effects. Expert Opin Investig Drugs. (2009) 18:585–600. doi: 10.1517/13543780902832661, PMID: 19388876 PMC3110777

[29] Hou X Zhou H. Research progress of cathepsin V. J Shenyang Med Coll. (2018) 20:464–6. doi: 10.16753/j.cnki.1008-2344.2018.05.023

[30] Edgington-Mitchell LE Rautela J Duivenvoorden HM Jayatilleke KM van der Linden WA Verdoes M. Cysteine cathepsin activity suppresses osteoclastogenesis of myeloid-derived suppressor cells in breast cancer. Oncotarget. (2015) 6:27008–22. doi: 10.18632/oncotarget.4714, PMID: 26308073 PMC4694970

[31] Jakoš T Pišlar A Pečar Fonović U Švajger U Kos J. Cysteine cathepsins L and X differentially modulate interactions between myeloid-derived suppressor cells and tumor cells. Cancer Immunol Immunother. (2020) 69:1869–80. doi: 10.1007/s00262-020-02592-x, PMID: 32372139 PMC11027625

[32] Tufail M Jiang C-H Li N. Altered metabolism in cancer: insights into energy pathways and therapeutic targets. Mol Cancer. (2024) 23:203. doi: 10.1186/s12943-024-02119-3, PMID: 39294640 PMC11409553

[33] Shang X Zhang S Ni J. Research progress on the role of cathepsin B in brain aging and the occurrence and development of alzheimer’s disease. Heredity. (2023) 45:212–20. doi: 10.16288/j.yczz.22-422, PMID: 36927647

[34] Hamon Y Legowska M Hervé V Dallet-Choisy S Marchand-Adam S Vanderlynden L. Neutrophilic cathepsin C is maturated by a multistep proteolytic process and secreted by activated cells during inflammatory lung diseases. J Biol Chem. (2016) 291:8486–99. doi: 10.1074/jbc.M115.707109, PMID: 26884336 PMC4861422

[35] Kain R Nackenhorst MC. A view on cathepsin C as a target for therapy in AAV. J Am Soc Nephrol. (2022) 33:875–8. doi: 10.1681/ASN.2022030309, PMID: 35396261 PMC9063890

[36] Cárcel-Trullols J Kovács AD Pearce DA. Cell biology of the NCL proteins: What they do and don’t do. Biochim Biophys Acta. (2015) 1852:2242–55. doi: 10.1016/j.bbadis.2015.04.027, PMID: 25962910

[37] Mitrović A Završnik J Mikhaylov G Knez D Pečar Fonović U Matjan Štefin P. Evaluation of novel cathepsin-X inhibitors *in vitro* and *in vivo* and their ability to improve cathepsin-B-directed antitumor therapy. Cell Mol Life Sci. (2022) 79:34. doi: 10.1007/s00018-021-04117-w, PMID: 34989869 PMC8738504

[38] Wang Y Zhao J Gu Y Wang H Jiang M Zhao S. Cathepsin H: Molecular characteristics and clues to function and mechanism. Biochem Pharmacol. (2023) 212:115585. doi: 10.1016/j.bcp.2023.115585, PMID: 37148981

[39] Aguda AH Panwar P Du X Nguyen NT Brayer GD Brömme D. Structural basis of collagen fiber degradation by cathepsin K. Proc Natl Acad Sci USA. (2014) 111:17474–9. doi: 10.1073/pnas.1414126111, PMID: 25422423 PMC4267343

[40] Sun Y-X Zhu B-J Tang L Sun Y Chen C Nadeem Abbas M. Cathepsin O is involved in the innate immune response and metamorphosis of. Antheraea pernyi. J Invertebrate Pathol. (2017) 150:6–14. doi: 10.1016/j.jip.2017.08.015, PMID: 28859880

[41] Senjor E Kos J Nanut MP. Cysteine cathepsins as therapeutic targets in immune regulation and immune disorders. Biomedicines. (2023) 11:476. doi: 10.3390/biomedicines11020476, PMID: 36831012 PMC9953096

[42] Smyth P Sasiwachirangkul J Williams R Scott CJ. Cathepsin S (CTSS) activity in health and disease - A treasure trove of untapped clinical potential. Mol aspects Med. (2022) 88:101106. doi: 10.1016/j.mam.2022.101106, PMID: 35868042

[43] Lecaille F Chazeirat T Saidi A Lalmanach G. Cathepsin V: Molecular characteristics and significance in health and disease. Mol Aspects Med. (2022) 88:101086. doi: 10.1016/j.mam.2022.101086, PMID: 35305807

[44] Li J Chen Z Kim G Luo J Hori S Wu C. Cathepsin W restrains peripheral regulatory T cells for mucosal immune quiescence. Sci Adv. (2023) 9:eadf3924. doi: 10.1126/sciadv.adf3924, PMID: 37436991 PMC10337914

[45] Gaffen SL. Signaling domains of the interleukin 2 receptor. Cytokine. (2001) 14:63–77. doi: 10.1006/cyto.2001.0862, PMID: 11356007

[46] Vizin T Christensen IJ Nielsen HJ Kos J. Cathepsin X in serum from patients with colorectal cancer: relation to prognosis. Radiol Oncol. (2012) 46:207–12. doi: 10.2478/v10019-012-0040-0, PMID: 23077459 PMC3472949

[47] Jakoš T Prunk M Pišlar A Kos J. Cathepsin X activity does not affect NK-target cell synapse but is rather distributed to cytotoxic granules. Int J Mol Sci. (2021) 22:13495. doi: 10.3390/ijms222413495, PMID: 34948293 PMC8707301

[48] Hou P-P Luo L-J Chen H-Z Chen Q-T Bian X-L Wu S-F. Ectosomal PKM2 promotes HCC by inducing macrophage differentiation and remodeling the tumor microenvironment. Mol Cell. (2020) 78:1192–1206.e10. doi: 10.1016/j.molcel.2020.05.004, PMID: 32470318

[49] Shi H Han X Sun Y Shang C Wei M Ba X. Chemokine (C-X-C motif) ligand 1 and CXCL2 produced by tumor promote the generation of monocytic myeloid-derived suppressor cells. Cancer Sci. (2018) 109:3826–39. doi: 10.1111/cas.13809, PMID: 30259595 PMC6272093

[50] Yang J Ren B Yang G Wang H Chen G You L. The enhancement of glycolysis regulates pancreatic cancer metastasis. Cell Mol Life Sci. (2020) 77:305–21. doi: 10.1007/s00018-019-03278-z, PMID: 31432232 PMC11104916

[51] Liu W-R Tian M-X Yang L-X Lin Y-L Jin L Ding Z-B. PKM2 promotes metastasis by recruiting myeloid-derived suppressor cells and indicates poor prognosis for hepatocellular carcinoma. Oncotarget. (2015) 6:846–61. doi: 10.18632/oncotarget.2749, PMID: 25514599 PMC4359260

[52] Noe JT Mitchell RA. MIF-dependent control of tumor immunity. Front Immunol. (2020) 11:609948. doi: 10.3389/fimmu.2020.609948, PMID: 33324425 PMC7724107

[53] Zhou Q Peng Y Ji F Chen H Kang W Chan L-S. Targeting of SLC25A22 boosts the immunotherapeutic response in KRAS-mutant colorectal cancer. Nat Commun. (2023) 14:4677. doi: 10.1038/s41467-023-39571-6, PMID: 37542037 PMC10403583

[54] Wang J Zhang X Li Z Li X Ma J Shen S. Identification of the interacting proteins with S100A8 or S100A9 by affinity purification and mass spectrometry. Zhong Nan Da Xue Xue Bao Yi Xue Ban. (2017) 42:365–73. doi: 10.11817/j.issn.1672-7347.2017.04.001, PMID: 28490692

[55] Deguchi A Watanabe-Takahashi M Mishima T Omori T Ohto U Arashiki N. Novel multivalent S100A8 inhibitory peptides attenuate tumor progression and metastasis by inhibiting the TLR4-dependent pathway. Cancer Gene Ther. (2023) 30:973–84. doi: 10.1038/s41417-023-00604-3, PMID: 36932197 PMC10021052

[56] Lv T Meng Y Liu Y Han Y Xin H Peng X. RNA nanotechnology: A new chapter in targeted therapy. Colloids Surf B Biointerfaces. (2023) 230:113533. doi: 10.1016/j.colsurfb.2023.113533, PMID: 37713955

[57] Zhang Z Zheng Y Chen Y Yin Y Chen Y Chen Q. Gut fungi enhances immunosuppressive function of myeloid-derived suppressor cells by activating PKM2-dependent glycolysis to promote colorectal tumorigene- sis. Exp Hematol Oncol. (2022) 11:88. doi: 10.1186/s40164-022-00334-6, PMID: 36348389 PMC9644472

[58] Liu Y Han Y Zhang Y Lv T Peng X Huang J. LncRNAs has been identified as regulators of Myeloid-derived suppressor cells in lung cancer. Front Immunol. (2023) 14:1067520. doi: 10.3389/fimmu.2023.1067520, PMID: 36817434 PMC9932034

[59] Wang D Zhao C Xu F Zhang A Jin M Zhang K. Cisplatin-resistant NSCLC cells induced by hypoxia transmit resistance to sensitive cells through exosomal PKM2. Theranostics. (2021) 11:2860–75. doi: 10.7150/thno.51797, PMID: 33456577 PMC7806469

[60] You L Wang Z Li H Shou J Jing Z Xie J. The role of STAT3 in autophagy. Autophagy. (2015) 11:729–39. doi: 10.1080/15548627.2015.1017192, PMID: 25951043 PMC4509450

[61] Yu S Pei S Zhang M Gao S Chen J Duan L. PKM2-mediated STAT3 phosphorylation promotes acute liver failure via regulating NLRP3-dependent pyroptosis. Commun Biol. (2024) 7:1694. doi: 10.1038/s42003-024-07227-w, PMID: 39722076 PMC11669718

[62] Wu J Lv T Liu Y Liu Y Han Y Liu X. The role of quercetin in NLRP3-associated inflammation. Inflammopharmacology. (2024) 32:3585–610. doi: 10.1007/s10787-024-01566-0, PMID: 39306817

[63] Zhong Z Wen Z Darnell JE. Stat3: a STAT family member activated by tyrosine phosphorylation in response to epidermal growth factor and interleukin-6. Science. (1994) 264:95–8. doi: 10.1126/science.8140422, PMID: 8140422

[64] Yu CL Meyer DJ Campbell GS Larner AC Carter-Su C Schwartz J. Enhanced DNA-binding activity of a Stat3-related protein in cells transformed by the Src oncoprotein. Science. (1995) 269:81–3. doi: 10.1126/science.7541555, PMID: 7541555

[65] Darnell JE Kerr IM Stark GR. Jak-STAT pathways and transcriptional activation in response to IFNs and other extracellular signaling proteins. Science. (1994) 264:1415–21. doi: 10.1126/science.8197455, PMID: 8197455

[66] Wingelhofer B Neubauer HA Valent P Han X Constantinescu SN Gunning PT. Implications of STAT3 and STAT5 signaling on gene regulation and chromatin remodeling in hematopoietic cancer. Leukemia. (2018) 32:1713–26. doi: 10.1038/s41375-018-0117-x, PMID: 29728695 PMC6087715

[67] House IG House CM Brennan AJ Gilan O Dawson MA Whisstock JC. Regulation of perforin activation and pre-synaptic toxicity through C-terminal glycosylation. EMBO Rep. (2017) 18:1775–85. doi: 10.15252/embr.201744351, PMID: 28808112 PMC5623865

[68] Demaria M Poli V. PKM2, STAT3 and HIF-1α: The Warburg’s vicious circle. JAKSTAT. (2012) 1:194–6. doi: 10.4161/jkst.20662, PMID: 24058770 PMC3670244

[69] Zhu Q Hong B Zhang L Wang J. Pyruvate kinase M2 inhibits the progression of bladder cancer by targeting MAKP pathway. J Cancer Res Ther. (2018) 14:S616–21. doi: 10.4103/0973-1482.187302, PMID: 30249877

[70] Park J-I. MAPK-ERK pathway. Int J Mol Sci. (2023) 24:9666. doi: 10.3390/ijms24119666, PMID: 37298618 PMC10253477

[71] Liu Y Cheng W Xin H Liu R Wang Q Cai W. Nanoparticles advanced from preclinical studies to clinical trials for lung cancer therapy. Cancer Nanotechnol. (2023) 14:28. doi: 10.1186/s12645-023-00174-x, PMID: 37009262 PMC10042676

[72] Chen Z Li Y Niu Y Zhang X Yu J Cui J. MEK1/2-PKM2 pathway modulates the immunometabolic reprogramming of proinflammatory allograft-infiltrating macrophages during heart transplant rejection. Transplantation. (2024) 108:1127–41. doi: 10.1097/TP.0000000000004899, PMID: 38238904 PMC11042528

[73] Kos J Jevnikar Z Obermajer N. The role of cathepsin X in cell signaling. Cell Adh Migr. (2009) 3:164–6. doi: 10.4161/cam.3.2.7403, PMID: 19262176 PMC2679876

[74] Zhao K Sun Y Zhong S Luo J-L. The multifaceted roles of cathepsins in immune and inflammatory responses: implications for cancer therapy, autoimmune diseases, and infectious diseases. biomark Res. (2024) 12:165. doi: 10.1186/s40364-024-00711-9, PMID: 39736788 PMC11687005

[75] Jechorek D Votapek J Meyer F Kandulski A Roessner A Franke S. Characterization of cathepsin X in colorectal cancer development and progression. Pathol Res Pract. (2014) 210:822–9. doi: 10.1016/j.prp.2014.08.014, PMID: 25442015

[76] Jevnikar Z Obermajer N Doljak B Turk S Gobec S Svajger U. Cathepsin X cleavage of the beta2 integrin regulates talin-binding and LFA-1 affinity in T cells. J Leukoc Biol. (2011) 90:99–109. doi: 10.1189/jlb.1110622, PMID: 21454358

[77] Breznik B Limbaeck Stokin C Kos J Khurshed M Hira VVV Bošnjak R. CCperi-arteriolar glioblastoma stem cell niches. J Mol Histol. (2018) 49:481–97. doi: 10.1007/s10735-018-9787-y, PMID: 30046941 PMC6182580

[78] Staudt ND Aicher WK Kalbacher H Stevanovic S Carmona AK Bogyo M. Cathepsin X is secreted by human osteoblasts, digests CXCL-12 and impairs adhesion of hematopoietic stem and progenitor cells to osteoblasts. Haematologica. (2010) 95:1452–60. doi: 10.3324/haematol.2009.018671, PMID: 20494937 PMC2930944

[79] Teller A Jechorek D Hartig R Adolf D Reißig K Roessner A. Dysregulation of apoptotic signaling pathways by interaction of RPLP0 and cathepsin X/Z in gastric cancer. Pathol Res Pract. (2015) 211:62–70. doi: 10.1016/j.prp.2014.09.005, PMID: 25433997

[80] Olson OC Joyce JA. Cysteine cathepsin proteases: regulators of cancer progression and therapeutic response. Nat Rev Cancer. (2015) 15:712–29. doi: 10.1038/nrc4027, PMID: 26597527

[81] Ben-Shmuel A Joseph N Barda-Saad M. Commentary: Integrins modulate T cell receptor signaling by constraining actin flow at the immunological synapse. Front Immunol. (2018) 9:2110. doi: 10.3389/fimmu.2018.02110, PMID: 30283450 PMC6157412

[82] Jevnikar Z Obermajer N Kos J. Cysteine protease-mediated cytoskeleton interactions with LFA-1 promote T-cell morphological changes. Cell Motil Cytoskeleton. (2009) 66:1030–40. doi: 10.1002/cm.20413, PMID: 19670215

[83] Skvarc M Stubljar D Kopitar AN Jeverica S Tepes B Kos J. Inhibition of cathepsin X enzyme influences the immune response of THP-1 cells and dendritic cells infected with Helicobacter pylori. Radiol Oncol. (2013) 47:258–65. doi: 10.2478/raon-2013-0043, PMID: 24133391 PMC3794882

[84] Obermajer N Svajger U Bogyo M Jeras M Kos J. Maturation of dendritic cells depends on proteolytic cleavage by cathepsin X. J Leukoc Biol. (2008) 84:1306–15. doi: 10.1189/jlb.0508285, PMID: 18701767 PMC3252843

[85] Feng J Li J Wu L Yu Q Ji J Wu J. Emerging roles and the regulation of aerobic glycolysis in hepatocellular carcinoma. J Exp Clin Cancer Res. (2020) 39:126. doi: 10.1186/s13046-020-01629-4, PMID: 32631382 PMC7336654

[86] Jiang H Liu C Shao Z. Research progress on the regulatory role of PKM2 in tumor cell metabolism. Biomed Eng Clin Med. (2017) 21:564–9. doi: 10.13339/j.cnki.sglc.20170906.025

[87] Chen M Liu H Li Z Ming AL Chen H. Mechanism of PKM2 affecting cancer immunity and metabolism in Tumor Microenvironment. J Cancer. (2021) 12:3566–74. doi: 10.7150/jca.54430, PMID: 33995634 PMC8120184

[88] Zhou T Pan J Liu C Liu D Yan C Song H. Regulation of mitochondrial biogenesis and energy metabolism in cardiomyocytes by pyruvate kinase M2 isoform. J Clin Military Med. (2024) 52:598–601. doi: 10.16680/j.1671-3826.2024.06.12

[89] Wang J-Z Zhu W Han J Yang X Zhou R Lu H-C. The role of the HIF-1α/ALYREF/PKM2 axis in glycolysis and tumorigene- sis of bladder cancer. Cancer Commun (Lond). (2021) 41:560–75. doi: 10.1002/cac2.12158, PMID: 33991457 PMC8286140

[90] Zeng C Wu J Li J. Pyruvate kinase M2: A potential regulator of cardiac injury through glycolytic and non-glycolytic pathways. J Cardiovasc Pharmacol. (2024) 84:1–9. doi: 10.1097/FJC.0000000000001568, PMID: 38560918 PMC11230662

[91] Zhu S Guo Y Zhang X Liu H Yin M Chen X. Pyruvate kinase M2 (PKM2) in cancer and cancer therapeutics. Cancer Lett. (2021) 503:240–8. doi: 10.1016/j.canlet.2020.11.018, PMID: 33246091

[92] Wang C Chen J Ju J Zhu X Wang C. Research on the mechanism of PKM2 promoting tumor development. Biotechnol Adv. (2022) 12:411–8. doi: 10.19586/j.2095-2341.2021.0181

[93] Vizovišek M Fonović M Turk B. Cysteine cathepsins in extracellular matrix remodeling: Extracellular matrix degradation and beyond. Matrix Biol. (2019) 75–76:141–59. doi: 10.1016/j.matbio.2018.01.024, PMID: 29409929

[94] Zhang W Wang S Wang Q Yang Z Pan Z Li L. Overexpression of cysteine cathepsin L is a marker of invasion and metastasis in ovarian cancer. Oncol Rep. (2014) 31:1334–42. doi: 10.3892/or.2014.2967, PMID: 24402045

[95] Pan T Jin Z Yu Z Wu X Chang X Fan Z. Cathepsin L promotes angiogenesis by regulating the CDP/Cux/VEGF-D pathway in human gastric cancer. Gastric Cancer. (2020) 23:974–87. doi: 10.1007/s10120-020-01080-6, PMID: 32388635 PMC7567730

[96] Zhang R Ruan Y Zhao Y Jin F Yang M Zhai Z. Targeting cathepsin L in the regulation of apoptosis in peripheral T-cell lymphoma. Mol Cell Toxicol. (2024) 20:541–52. doi: 10.1007/s13273-023-00359-w

[97] Du X Ding L Huang S Li F Yan Y Tang R. Cathepsin L promotes chemresistance to neuroblastoma by modulating serglycin. Front Pharmacol. (2022) 13:920022. doi: 10.3389/fphar.2022.920022, PMID: 36133820 PMC9484481

[98] Pišlar A Perišić Nanut M Kos J. Lysosomal cysteine peptidases - Molecules signaling tumor cell death and survival. Semin Cancer Biol. (2015) 35:168–79. doi: 10.1016/j.semcancer.2015.08.001, PMID: 26255843

[99] Khaket TP Kwon TK Kang SC. Cathepsins: Potent regulators in carcinogenesis. Pharmacol Ther. (2019) 198:1–19. doi: 10.1016/j.pharmthera.2019.02.003, PMID: 30763594

[100] Huang X Li Z Huang Y Zhang Q Cui Y Shi X. Vimentin intermediate filaments coordinate actin stress fibers and podosomes to determine the extracellular matrix degradation by macrophages. Dev Cell. (2025) 60(12):1669–85.e6. doi: 10.1016/j.devcel.2025.01.016, PMID: 39952241

[101] Sevenich L Schurigt U Sachse K Gajda M Werner F Müller S. Synergistic antitumor effects of combined cathepsin B and cathepsin Z deficiencies on breast cancer progression and metastasis in mice. Proc Natl Acad Sci USA. (2010) 107:2497–502. doi: 10.1073/pnas.0907240107, PMID: 20133781 PMC2823914

